# Spontaneous regression of stage IV advanced diffuse large B-cell lymphoma in an person living with HIV: a case report and literature review

**DOI:** 10.3389/fonc.2026.1779897

**Published:** 2026-04-29

**Authors:** Wei Zhang, Changgang Deng, Qisui Li, Jing Yuan

**Affiliations:** Division of Infectious Diseases, Chongqing Public Health Medical Center, Chongqing, China

**Keywords:** combination antiretroviral therapy, diffuse large B-cell lymphoma, HIV, liver, spontaneous regression, stage IV

## Abstract

**Background:**

HIV-associated diffuse large B-cell lymphoma (DLBCL) is an aggressive malignancy with poor outcomes despite combination antiretroviral therapy (cART). Spontaneous regression in advanced-stage disease is exceptionally rare.

**Case description:**

A 66-year-old male with stage IV, non-germinal center DLBCL involving the liver achieved complete radiographic resolution of multifocal hepatic lesions without cytotoxic therapy following cART initiation. Remission has been sustained for over 36 months. Effective viral suppression and CD4+ T-cell recovery (from 200 to 571 cells/μL) were temporally correlated with tumor regression.

**Conclusion:**

Spontaneous regression of stage IV diffuse large B−cell lymphoma (DLBCL) is exceedingly rare among individuals with HIV infection. This case underscores the critical role of immune reconstitution via cART in treating HIV−associated lymphoma. Therefore, it is recommended to initiate cART as early as possible in HIV−positive lymphoma patients to restore immune function, which can positively influence lymphoma management.

## Introduction

Acquired Immunodeficiency Syndrome (AIDS) remains a critical public health issue, representing a significant threat to global health. With the long-term effectiveness of cART in treating HIV/AIDS patients, life expectancy has markedly improved, and AIDS has transitioned into a chronic, manageable condition (1). Concurrently, there has been an increasing prevalence of non-HIV-defining illnesses, including malignancies, which now exhibit a mortality rate twice that of HIV-defining diseases (2). Since 2017, the incidence of HIV-associated non-Hodgkin lymphoma has exceeded that of Kaposi’s sarcoma, emerging as the most common HIV-related malignancy, with an incidence ranging from 100 to 300 per 100,000 individuals (3). Among these, DLBCL constitutes the most frequent pathological subtype of HIV-related non-Hodgkin lymphoma (4), and has become a major determinant of quality of life and prognosis for people living with HIV in the post-cART era (5).

Research indicates that (6, 7) the pathogenesis of DLBCL in individuals with HIV is largely attributable to HIV-mediated immune system damage. Upon entry into the human body, the virus specifically targets CD4^+^ T lymphocytes, resulting in severe immune dysfunction and diminished immune surveillance. This immunodeficiency state predisposes B lymphocytes to aberrant proliferation and malignant transformation, thereby initiating DLBCL. Furthermore, HIV promotes co-infection with potential oncogenic viruses—such as Epstein-Barr virus (EBV), HHV-8, hepatitis B virus (HBV), and hepatitis C virus (HCV)—which further elevates the risk of lymphoma development (8–10).

In clinical practice, the treatment of HIV-associated DLBCL typically involves a comprehensive approach similar to that used for non-HIV-infected populations, primarily based on chemotherapy and immunotherapy. For HIV-positive patients, cART is also integrated to suppress HIV viral load and improve immune function. Nevertheless, even with aggressive treatment, the prognosis for patients with HIV-associated DLBCL remains poor, particularly for those with stage IV disease, who tend to have worse outcomes (11, 12). Within this group of stage IV DLBCL patients living with HIV, cases of spontaneous remission are exceedingly rare, and reports in the literature are scarce.

This case report details a patient with HIV-positive stage IV DLBCL, focusing on the diagnostic and therapeutic course as well as the observed spontaneous tumor regression. By integrating a literature review, the report provides an in-depth analysis of this rare phenomenon, highlighting both commonalities and variations. The findings are intended to offer novel perspectives and practical references for the clinical management of HIV-associated DLBCL, ultimately aiming to improve the understanding and approach to disease outcomes in this specific patient population.

## Case presentation

### Patient information

**Figure 1:**
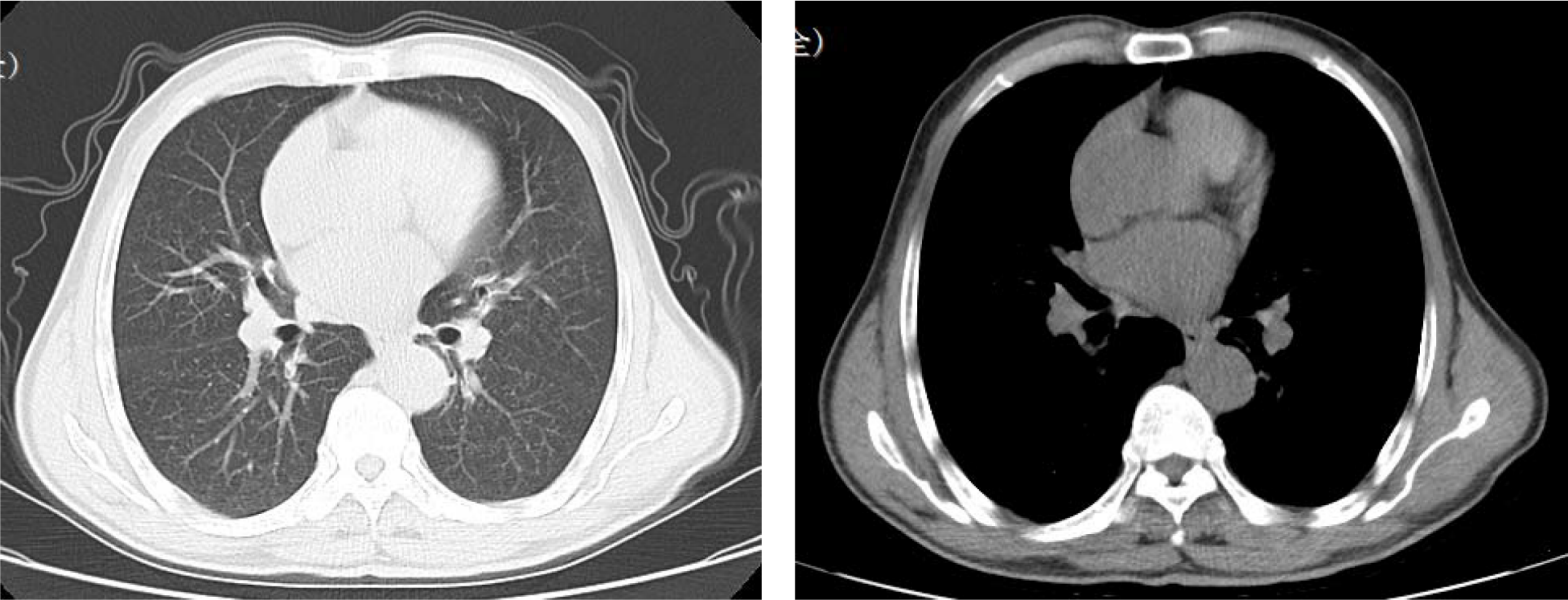
Chest computed tomography (CT) scan on November 26, 2021 showed increased pulmonary markings with scattered nodules.

**Figure 2:**
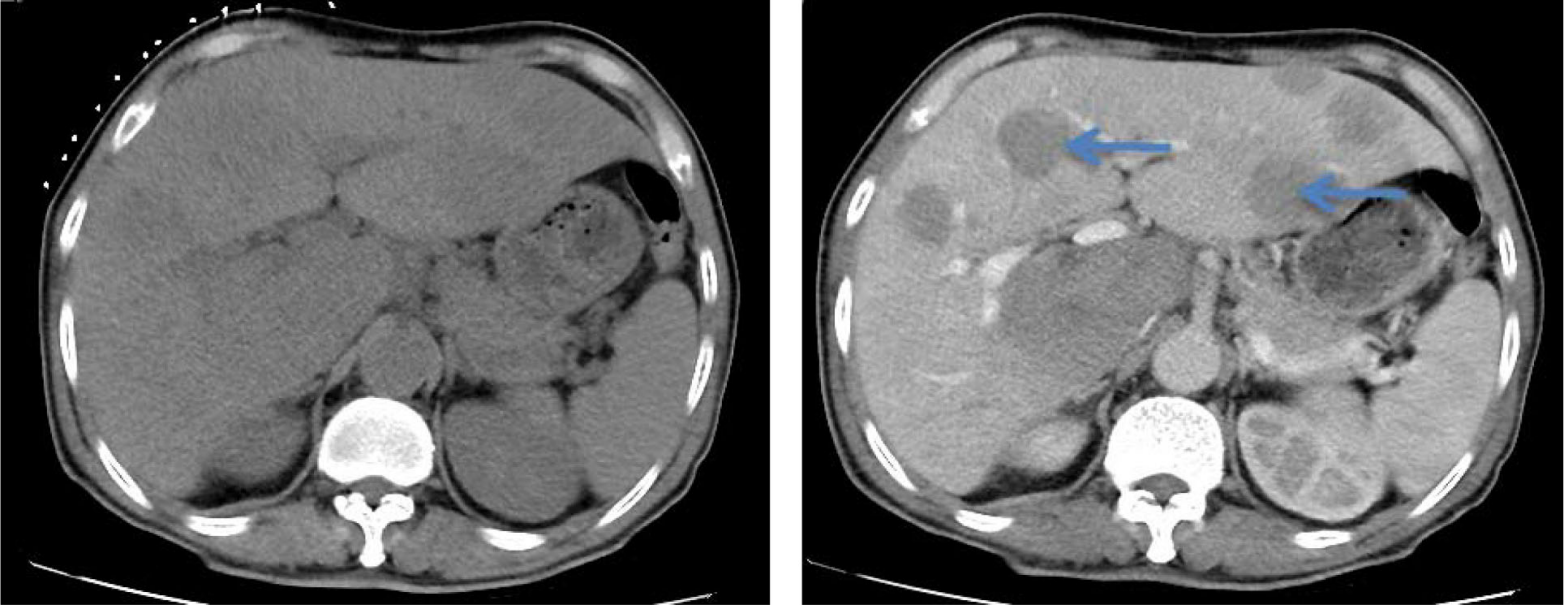
Abdominal enhanced CT scan on November 26, 2021 identified multiple hepatic lesions. The blue arrow indicates a liver mass lesion.

A 66-year-old male patient presented with a one-month history of cough and was found to have hepatic space-occupying lesions on imaging two weeks prior to admission. The patient was referred to our hospital following initial evaluation at a hospital outside our center, where chest CT revealed increased lung markings with scattered nodules and abdominal CT demonstrated multiple hepatic lesions. The patient was referred to our facility after an initial assessment at a hospital outside our center. Chest computed tomography (CT) showed increased pulmonary markings with scattered nodules, while abdominal CT identified multiple hepatic lesions (**Figures 1**, **2**). The patient reported an unintentional weight loss of approximately 3 kg over the past month. His medical history included a 30-year smoking history (20 cigarettes/day) and heterosexual promiscuity, with no history of alcohol abuse or viral hepatitis. Physical examination revealed stable vital signs (T 36.6 °C, P 86 bpm, R 21 bpm, BP 101/65 mmHg, SpO_2_ 94%) without palpable superficial lymphadenopathy or edema.

### Clinical findings

**Figure 3:**
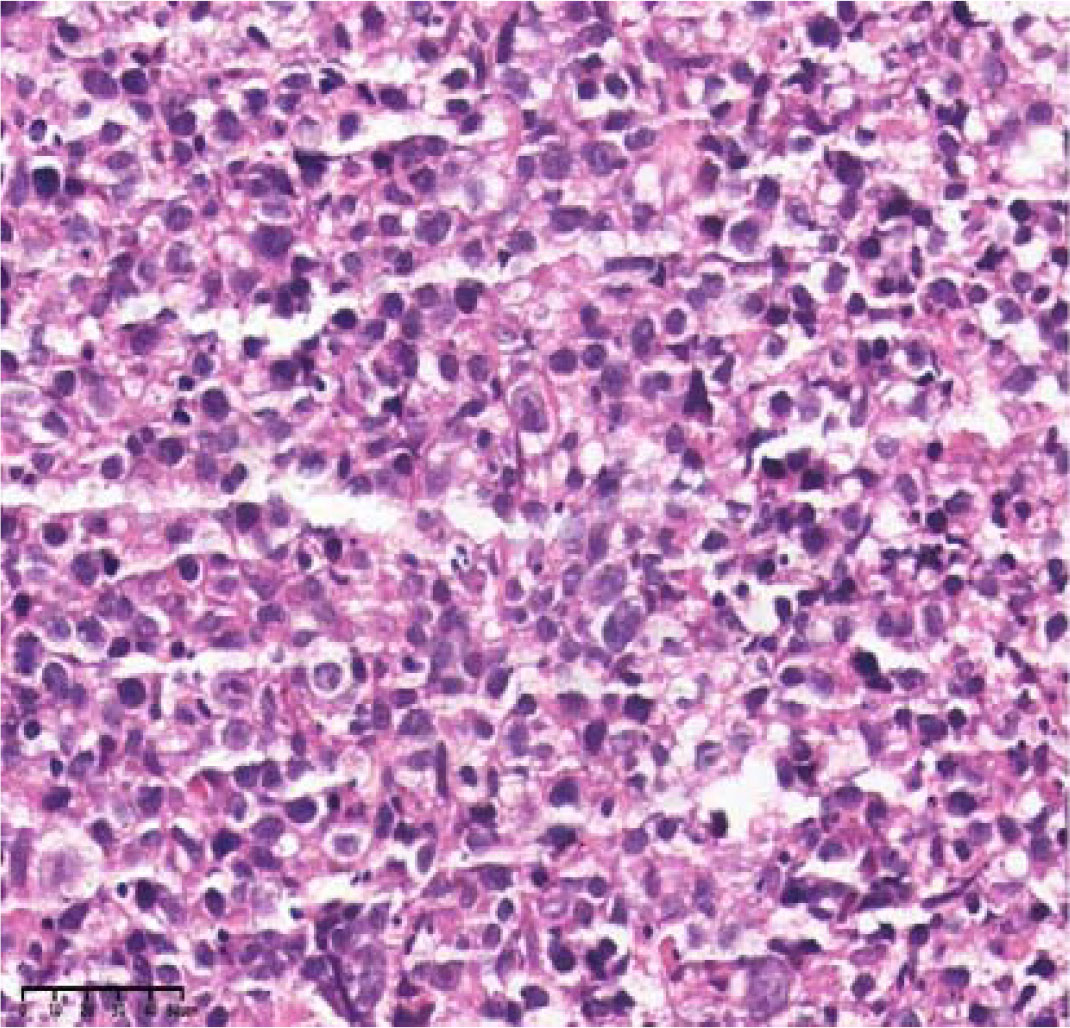
H&E stain, 40×. The tumor cells are medium to large lymphocytes with large nuclei, prominent nucleoli, and abundant cytoplasm.

**Figure 4:**
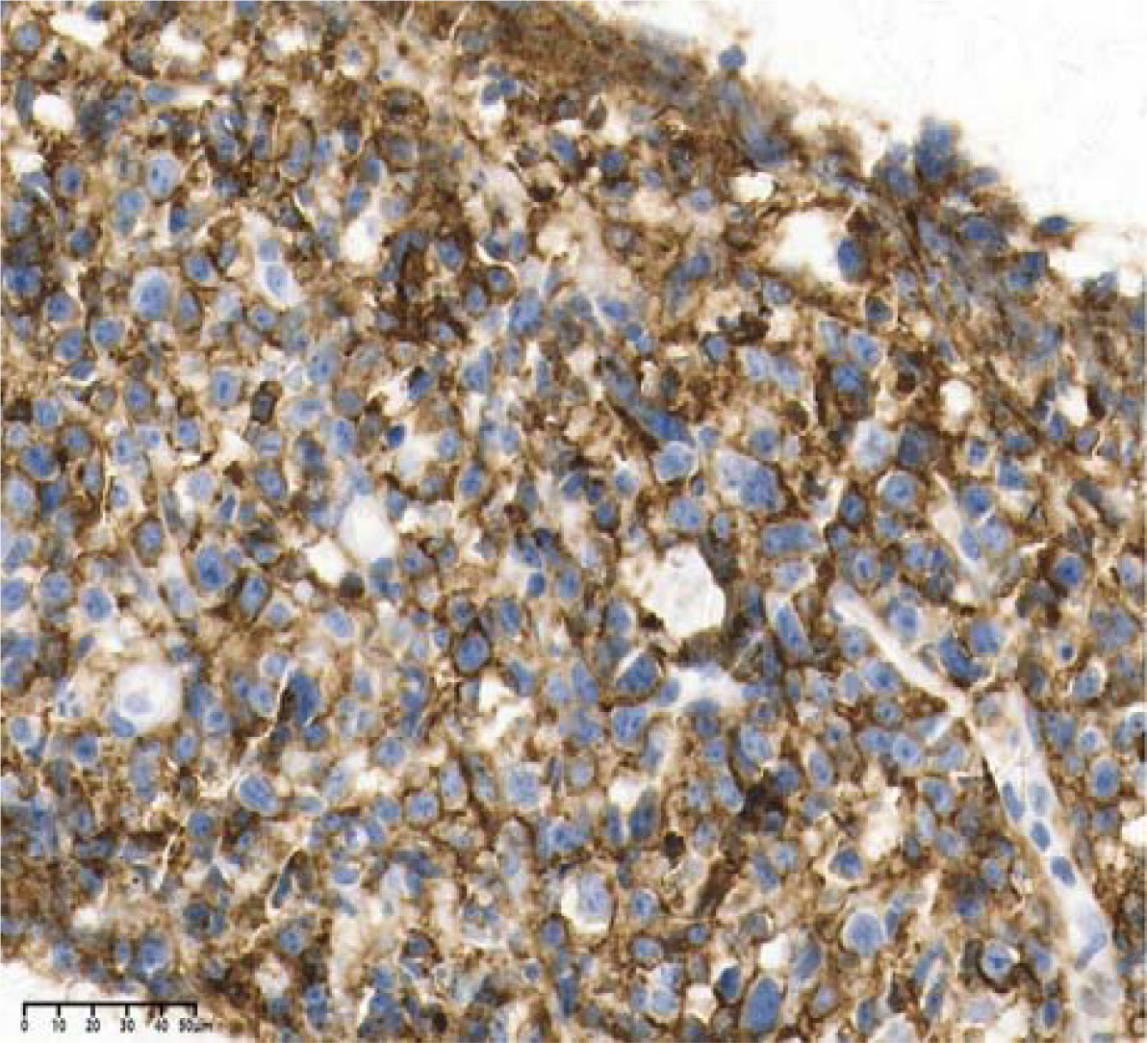
CD20(+), 40×.

**Figure 5:**
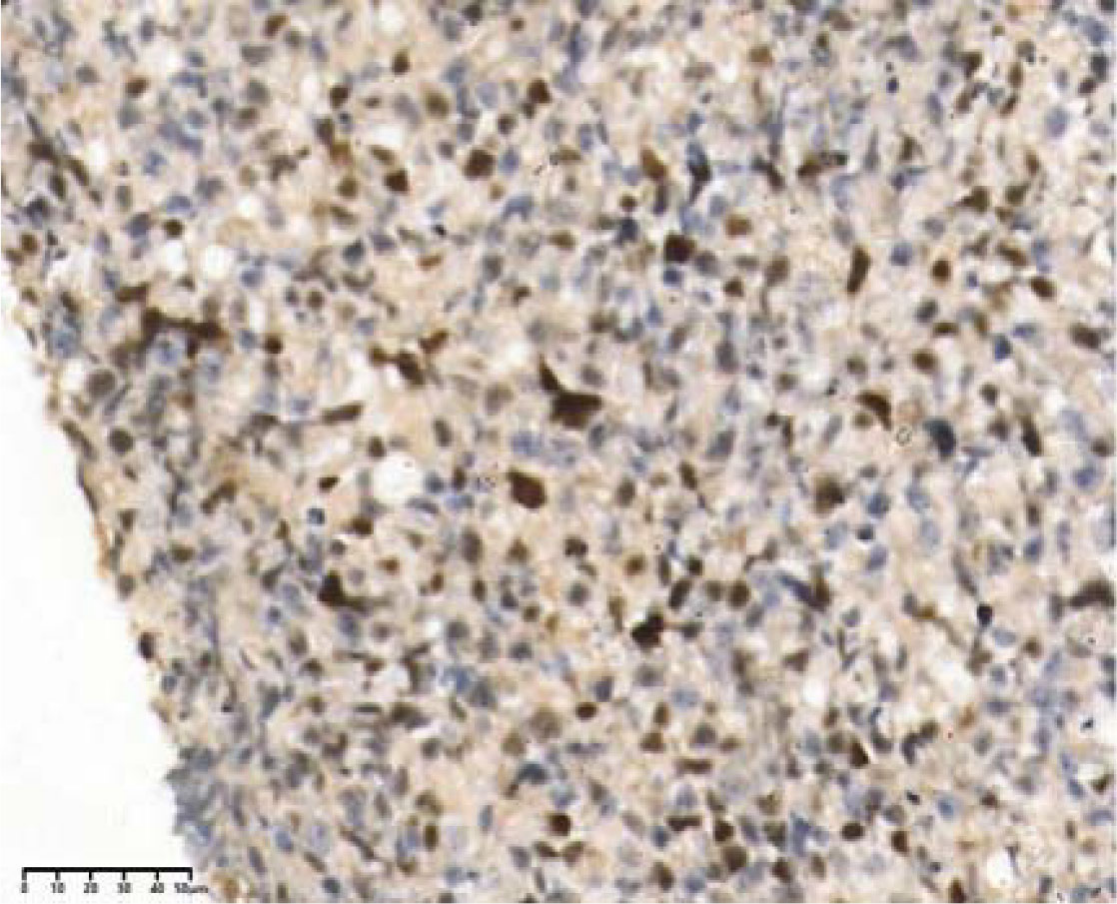
C-myc(+), 40×.

**Figure 6:**
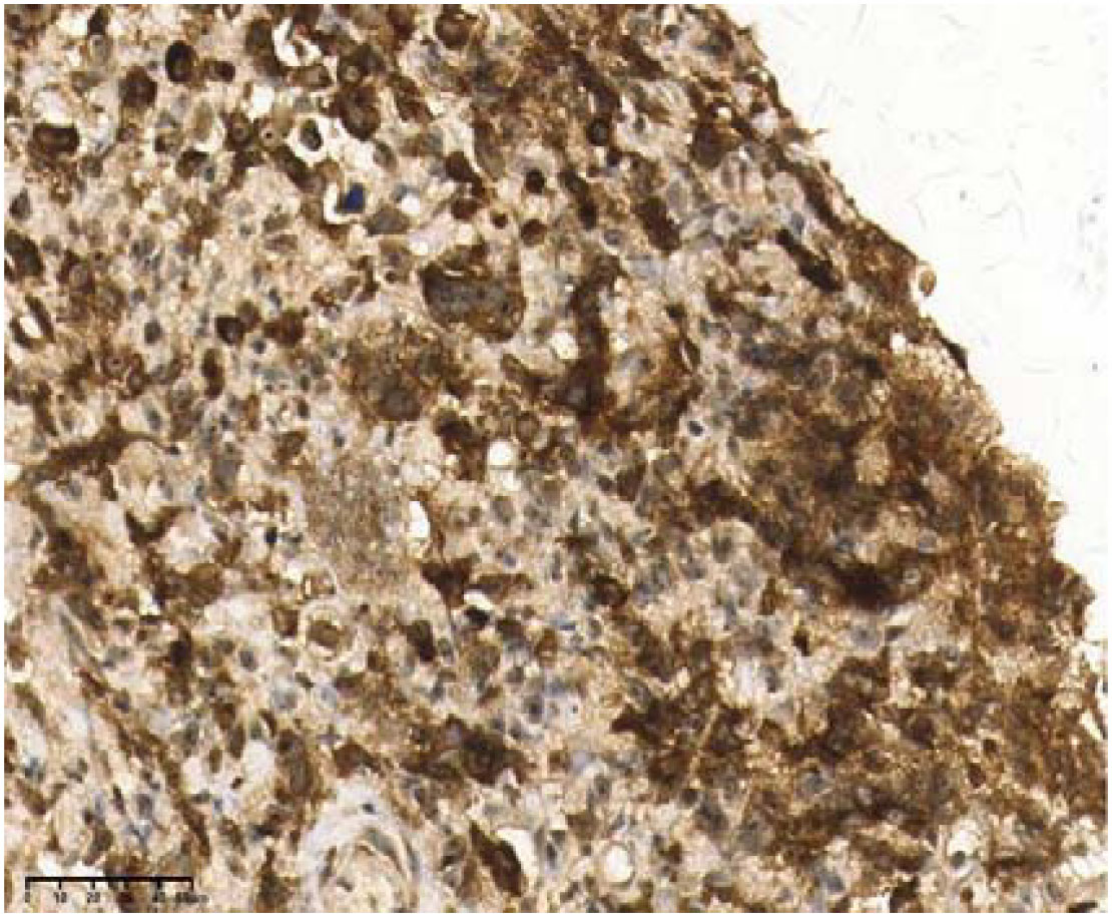
Bcl-2(+), 40×.

**Figure 7:**
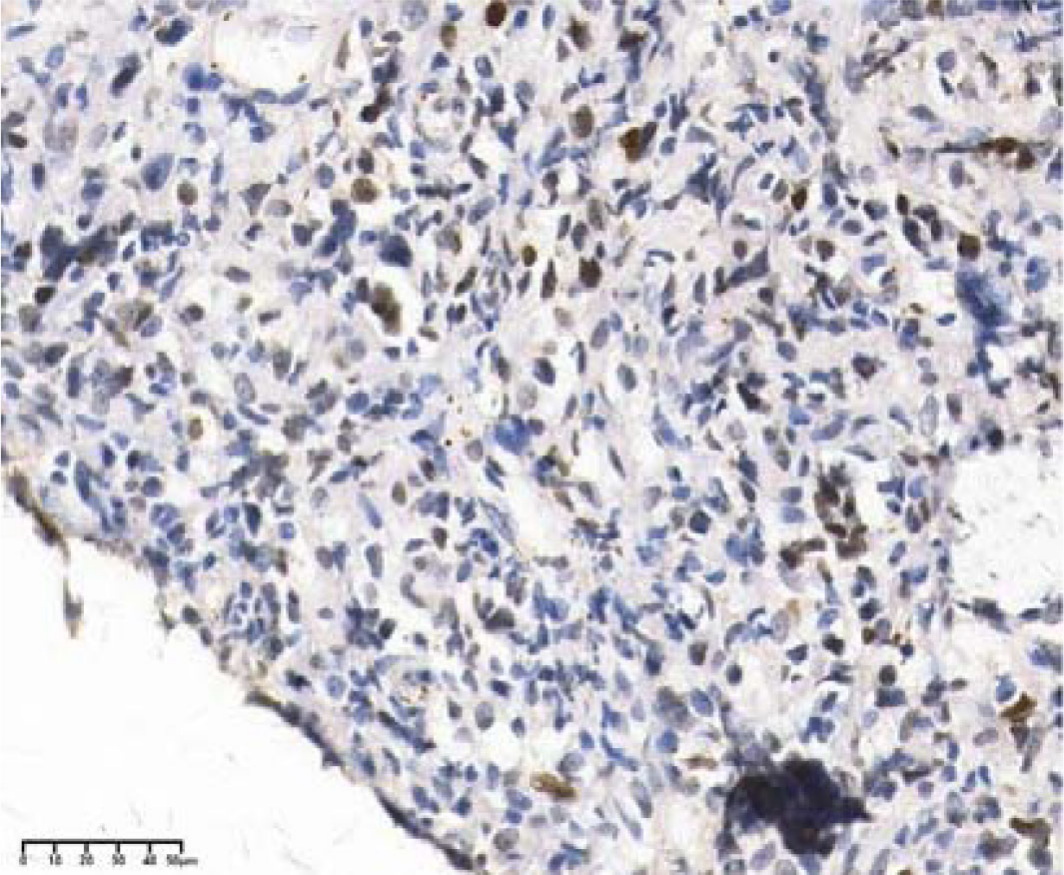
Bcl-6(+), 40×.

**Figure 8:**
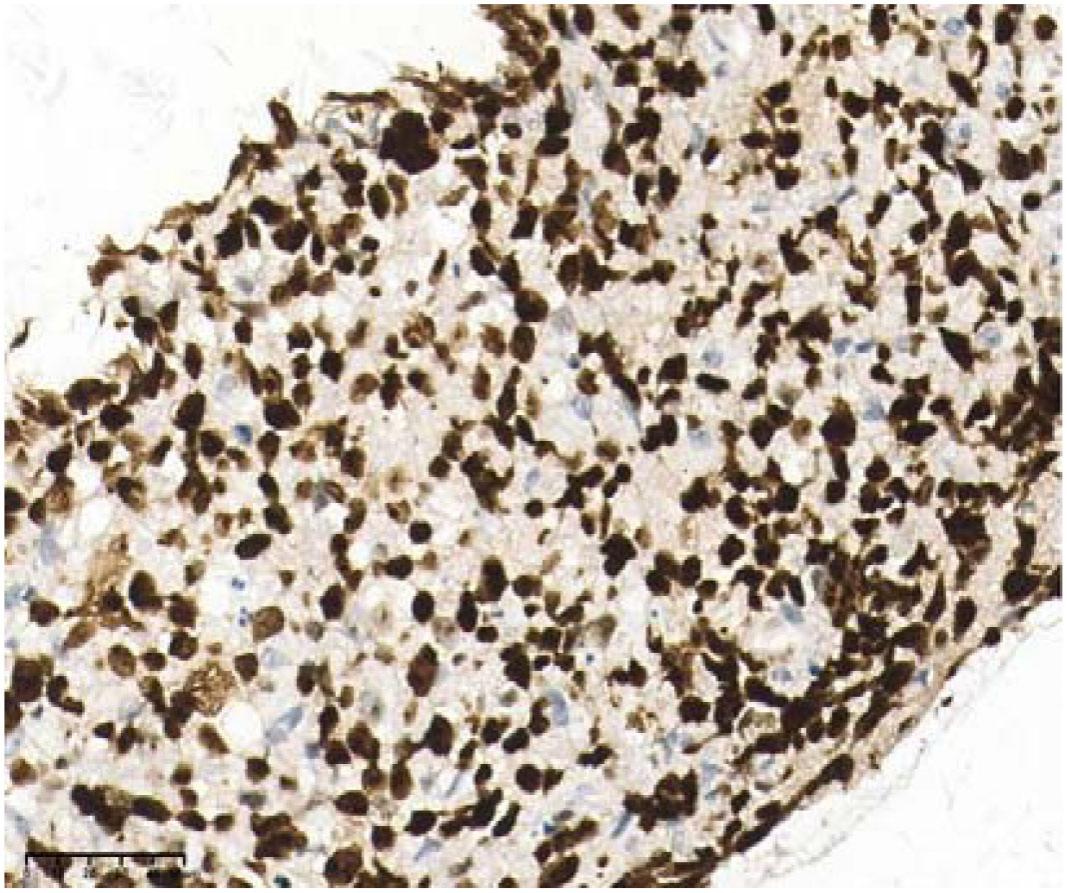
Ki67: 80%, 40×.

Upon admission, the patient experienced recurrent fever with a peak temperature of 39.8 °C. Laboratory investigations demonstrated anemia (Hb 88 g/L), thrombocytopenia (92×10^9^/L), elevated inflammatory markers (CRP 35.08 mg/L, PCT 0.2 ng/mL), and significant immunosuppression (CD4^+^ T-cell count 200 cells/μL, CD4/CD8 ratio 0.42). HIV RNA viral load was markedly elevated (3.22×10^6^ copies/mL), with concomitant EBV viremia (1.38×10^5^ copies/mL). Imaging studies revealed diffuse pulmonary nodules(the dominant pulmonary nodule measured 1.8 cm), bilateral axillary and mediastinal lymphadenopathy, and multiple hepatic lesions suspicious(the largest one measures approximately 5 cm in diameter) for neoplastic or infectious etiology. Histopathological examination of CT-guided liver biopsy demonstrated diffuse infiltration of atypical lymphoid cells with immunophenotypic features consistent with HIV-associated DLBCL) non-germinal center B-cell-like (non-GCB) subtype (CD20^+^, CD10^-^,Bcl-2^+^, Bcl-6^+^, C-myc^+^,MUM-1^+^, HHV-8^-^,Ki-67 80%), Two senior pathologists (with senior professional titles and over 20 years of experience in hematological pathology diagnosis) provided a second pathological review for this case. They are from the Pathology Department of Chongqing Cancer Hospital and both agreed with the above pathological diagnosis (**Figures 3**–**8**).

### Diagnostic assessment

The patient was diagnosed with stage IV HIV-associated DLBCL (non-GCB type, IPI score 5, high-risk group), complicated by hypoalbuminemia (25.8 g/L) and moderate anemia. Despite comprehensive counseling, the patient declined standard chemotherapy regimens, PET-CT staging, and invasive procedures including bone marrow and lumbar puncture.

### Therapeutic intervention

**Figure 9:**
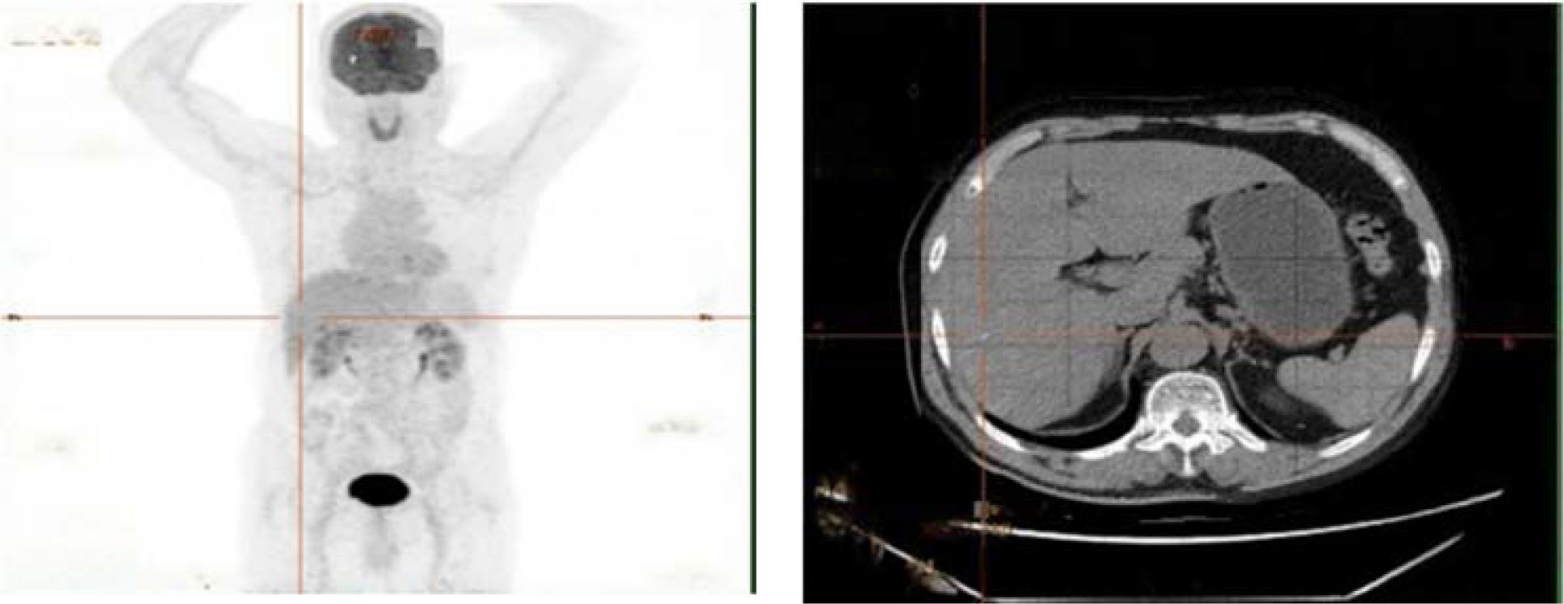
2022-09–24 PET-CT demonstrated complete resolution of previously documented hepatic lesions and nodal involvement, with no evidence of FDG-avid disease.

cART with Albuvirtide/lamivudine/dolutegravir was initiated on December 2, 2021,the patient’s constitutional symptoms (fever, night sweats) resolved within 4 weeks. The regimen was subsequently switched to bictegravir/emtricitabine/tenofovir alafenamide during outpatient follow-up. Notably, when the patient re-presented in September 2022 for chemotherapy initiation, PET-CT demonstrated complete resolution of previously documented hepatic lesions and nodal involvement, with no evidence of FDG-avid disease (**Figure 9**).

### Follow-up and outcomes

Serial monitoring revealed immunological recovery (CD4^+^ T-cells 571/μL, CD4/CD8 ratio 0.78) and sustained virological suppression (HIV RNA <50 copies/mL). Over 36 months of continuous follow-up, the patient maintained complete remission without disease recurrence, demonstrating an exceptional case of spontaneous regression in advanced HIV-associated DLBCL following immune reconstitution through cART alone. This clinical course challenges conventional therapeutic paradigms and underscores the potential role of immune-mediated tumor control in select HIV-associated malignancies.

## Discussion

Spontaneous tumor regression is a rare phenomenon, with an estimated incidence of approximately 1 in 60,000 to 100,000 cases (13). It is defined as the complete or partial disappearance of a tumor, occurring either without treatment or after therapy considered inadequate to significantly impact the malignant disease (14). This phenomenon is observed more frequently in certain tumor types, including neuroblastoma, testicular tumors, renal cell carcinoma, melanoma, and lymphoma (15). Nevertheless, data on spontaneous regression in HIV-positive patients remain limited.

Most patients with HIV-associated DLBCL are diagnosed at an advanced stage, often complicated by severe immunosuppression and multiple opportunistic infections, leading to a generally poor prognosis (16). Although the present case also involved stage IV disease, the patient experienced spontaneous tumor regression in the absence of systematic chemotherapy, thereby deviating from the typical clinical course of progressive deterioration in advanced DLBCL. Therapeutically (17), standard guidelines recommend a combined regimen of chemotherapy, immunotherapy, and cART to optimally suppress tumor growth and enhance immune recovery. In contrast, this patient—owing to physical debilitation and apprehensions regarding chemotherapy—declined cytotoxic treatment and received only cART, yet achieved a positive outcome. This contrasts with the conventional understanding that intensive antitumor therapy is essential for prognostic improvement. With regard to prognosis, prior studies indicate that even with standard treatment, stage IV HIV-associated DLBCL is associated with low five-year survival and a high risk of relapse (18). Notably, following spontaneous regression, this patient exhibited no evidence of recurrence, achieved near-normal immune reconstitution, and reported substantially improved quality of life—thereby representing a rare example of clinical cure that surpasses the expected outcomes for this patient group. Investigating such exceptional cases may provide valuable insights into the biology of HIV-related DLBCL and reveal potential avenues for therapeutic innovation.

Although spontaneous tumor regression has been reported in HIV-associated malignancies such as plasmablastic lymphoma and primary effusion lymphoma, documented cases in patients with HIV-positive stage IV diffuse large B-cell lymphoma (DLBCL) remain scarce. We conducted a PubMed search using the keywords: HIV, diffuse large B-cell lymphoma, stage IV, cART, and spontaneous regression, which identified four relevant case reports (19–22). These cases, along with the present case (Case 1), are summarized in **Table 1**. All five patients were male, with a mean age of 54.2 years, and each was diagnosed with stage IV lymphoma. Following the initiation of cART, all patients demonstrated a rise in CD4^+^ T-cell counts, effective control of HIV viral load, and attained complete remission of lymphoma without receiving chemotherapy or any other antitumor treatment. During a follow-up period of up to 96 months, none of the patients experienced disease relapse.

**Table 1:** Summary of characteristics of HIV-associated stage IV DLBCL with spontaneous regression.

Case number	Gender	Age	CD4 at diagnosis (cells/μL)	CD4 at resolution (cells/μL)	HIV RNA at diagnosis (copies/ml)	HIV RNA at resolution (copies/ml)	Site	Stage	Oncogenic virus	cART	Follow-up (months)	References
1	Male	66	200	571	3.22E+06	<50	Liver	IVB	EBV	3TC/DTG/ABT/Biktarvy	36	Our present case
2	Male	65	70	147	227466	241	–	IVB	No	No	24	(19)
3	Male	44	69	194	510327	Undetectable	Liver	IVB	No	Zidovudine/Lamivudine + Lopinavir/Ritonavir	96	(20)
4	Male	60	Data not available	Data not available	Data not available	Data not available	Liver	IVA	HCV	Data not available	60	(21)
5	Male	32	2	142	148	Undetectable	Colon	IVB	EBV	TDF/FTC/RAL	12	(22)

The observed alterations in immune function in patients with HIV-associated DLBCL are intimately related to the spontaneous regression of the tumor. Following effective antiviral therapy, immune reconstitution occurs, accompanied by the restoration of functional capacities in immune cells, including T lymphocytes and natural killer (NK) cells, which subsequently re−establish their roles in immune surveillance and tumor cell elimination. Studies indicate (23–25) that immune cells can recognize antigenic peptide–MHC complexes on the surface of tumor cells, thereby activating cytotoxic T lymphocytes and releasing molecules such as perforin and granzymes, which induce tumor cell apoptosis. Simultaneously, NK cells contribute through the direct lysis of tumor cells or the secretion of cytokines that modulate the immune response and suppress tumor progression. In the cases examined, progressive enhancement of immune function resulted in heightened immune pressure on tumor cells, restricting their proliferation and ultimately giving rise to spontaneous regression. These findings underscore the pivotal role of the host immune system in the antitumor process.

The tumor microenvironment (TME) plays a fundamental role in tumor cell growth, proliferation, and metastasis. In patients with HIV-associated DLBCL, HIV infection promotes a state of chronic inflammation that disrupts the TME, resulting in elevated secretion of pro-tumor cytokines—such as tumor necrosis factor-alpha (TNF-α) and interleukin-6 (IL-6)—that foster a conducive milieu for tumor progression (26, 27). In the reported cases, during chemotherapy-free follow-up, the TME underwent gradual modification concurrent with overall clinical improvement. Moreover, immune reconstitution following cART facilitated the infiltration of immune cells into tumor tissues, recalibrating the immune balance within the TME and promoting a shift from a pro-tumor to an anti-tumor phenotype—a transition potentially linked to spontaneous tumor regression.

It is critically important to emphasize that the standard of care for HIV-associated DLBCL is intensive immunochemotherapy (e.g., R-CHOP or R-EPOCH) combined with cART, as recommended by international guidelines (28, 29). People living with HIV (PLWH) should be considered suitable candidates for full-dose immunochemotherapy, autologous stem cell transplantation, and even CAR-T cell therapies, with outcomes comparable to HIV-negative individuals when HIV is well controlled. The prognosis of HIV-associated lymphomas has improved dramatically in the cART era, but this improvement depends on timely and adequate anti−lymphoma treatment. Our case represents a rare exception; spontaneous regression following cART alone is not typical and should not be used to justify deferring or withholding standard cytotoxic therapy. Clinicians must continue to offer guideline−based immunochemotherapy to eligible patients.

This extremely rare case does NOT contradict standard guidelines; intensive immunochemotherapy plus cART remains the standard of care. This observation suggests a potential immune-mediated remission subset in PLWH with DLBCL, warranting further biological study.

## Conclusion

In summary, we report a case of spontaneous regression of stage IV with hepatic involvement in an PLWH, with no recurrence documented over a 3-year follow-up period. Spontaneous resolution of lymphoma in the context of HIV infection remains exceedingly rare, with only a few cases reported in the literature. This case highlights the critical role of immune reconstitution through cART in the management of HIV-associated lymphoma, thereby supporting the initiation of antiretroviral therapy as early as possible in such patients, while continually evaluating their eligibility for chemotherapy within a tailored treatment strategy.

## Data Availability

The original contributions presented in the study are included in the article/supplementary material. Further inquiries can be directed to the corresponding author.
